# Living Apart Together and Older Adults’ Mental Health in the United Kingdom

**DOI:** 10.1093/geronb/gbae192

**Published:** 2024-12-03

**Authors:** Yang Hu, Rory Coulter

**Affiliations:** Department of Sociology, Lancaster University, Lancaster, UK; Department of Geography, University College London, London, UK; (Social Sciences Section)

**Keywords:** Family diversity, Gender, Life course, Relationship

## Abstract

**Objectives:**

Living apart together (LAT)—intimate partners living in separate households—is a common partnership type among older adults. Although the mental health benefits of intimate partnerships are widely documented, how LAT relates to older adults’ mental health remains understudied.

**Methods:**

Analyzing Waves 3–13 (2011–2023) of the United Kingdom Household Longitudinal Study, we use fixed effects models to examine (a) how older adults’ mental health varies with LAT, marriage, cohabitation, and singlehood (never married, widowed, divorced/separated) and (b) how transitions into and out of LAT, compared with marriage and cohabitation, relate to older adults’ mental health.

**Results:**

Overall, older adults have better mental health when LAT than when single, but little difference in mental health is found across LAT, cohabitation, and marital partnerships. Whereas older singles moving into LAT experience mental health improvements, those moving from LAT to singlehood suffer mental health declines. Although the mental health benefits of moving into LAT are smaller than those of entering cohabitation and particularly marriage, exiting LAT is associated with smaller mental health declines compared with exiting cohabitation and marriage. No statistically significant gender difference is found in the mental health benefits of LAT.

**Discussion:**

The findings underscore LAT as a key form of family diversity in later life. They problematize the long-held ideal of coresidence in couple relationships and its role in sustaining older adults’ mental health. They encourage researchers to go beyond the household as a default unit of analysis and examine interhousehold intimate connections in older adults’ lives.

Intimate partnerships play a crucial role in sustaining older adults’ well-being (de Jong Gierveld and Broese van Groenou, 2016; Manning and Brown, 2011; Rowe and Kahn, 1998, 2015; Switsers et al., 2023). As there is now much diversity in older adults’ partnership types (Benson and Coleman, 2016; Brown and Lin, 2012; Duncan et al., 2013; Gopinath et al., 2023; Manning and Brown, 2011; Sassler and Lichter, 2020), this study examines how an understudied type of partnership—living apart together (LAT) relationships—relates to older adults’ mental health. In LAT unions, partners do not permanently live together but instead maintain an intimate relationship while (sometimes) living in different households (Benson, 2015; Duncan and Phillips, 2011). Whereas for younger adults LAT is often a transitory stage preceding cohabitation and marriage, LAT is recognized to often be a more long-term and stable type of partnership in later life, which remains understudied as a crucial form of family diversity in the aging population (Connidis et al., 2017; Coulter and Hu, 2017). LAT allows older adults to balance interdependence and autonomy across their personal lives, which is particularly important given the growing complexity of family relations in later life, for example, with ex-spouses, adult children, and grandchildren following divorce or widowhood events (Benson and Coleman, 2016; Duncan, 2015; Karlsson and Borell, 2002).

There is a dearth of population-representative longitudinal research on the implications of LAT for older adults’ mental health. Most research on older adults’ partnerships and mental well-being has focused on co-residential relationships by comparing singlehood with cohabitation and marriage (e.g., Brown and Lin, 2012; Lin et al., 2019; Rapp and Stauder, 2020). Although an emerging line of research has begun to examine the mental health implications of LAT, this research has mostly analyzed cross-sectional data (Brown et al., 2022; Ciritel, 2022; Dush and Amato, 2005; Lewin, 2017; Liefbroer et al., 2015; Ross, 1995; Tai et al., 2014; Yucel and Latshaw, 2023). Despite providing valuable insights into the association between LAT and well-being, such cross-sectional analyses are unable to disentangle whether such associations arise from *selection* into LAT (possibly based on mental health status) or *the effect* of LAT on mental health. To overcome this limitation, we build on emerging longitudinal research exploring LAT’s effect on mental health in Germany and Australia (Evans et al., 2023; Rapp and Stauder, 2020; Ševčíková et al., 2021) by providing the first longitudinal analysis of the mental health implications of LAT for older adults in the United Kingdom.

Analyzing nationally representative data from the United Kingdom Household Longitudinal Study (UKHLS; 2011–2023, *N* = 93,885 observations of 15,237 older adults aged 60–85), we use fixed effects models to examine (a) how older adults’ mental health varies with LAT, cohabitation, marriage, and singlehood (never married, widowed, divorced/separated); (b) how transitions into and out of LAT, cohabitation, and marriage relate to changes in older adults’ mental health; and (c) gender differences in these associations. Our findings show that LAT is a crucial partnership type in later life that affects older adults’ mental health in ways that have hitherto been overlooked.

## Theoretical Considerations

### The Mental Health Benefits of Partnerships

It is widely known that couple relationships benefit older adults’ mental health (de Jong Gierveld and Broese van Groenou, 2016; Evans et al., 2023; Switsers et al., 2023). Several theoretical frameworks have been developed to explain these findings and to help understand how different types of couple relationships may convey differential mental health benefits. Notably, Rowe and Khan’s (1998, 2015) influential framework highlights how active engagement with life and interpersonal relationships that sustain exchanges of information, emotional support, and direct assistance are crucial factors contributing to successful aging. Relatedly, social convoy theory underscores the importance of intimate partnerships in providing older adults with health-enhancing forms of everyday companionship (Fuller et al., 2020). The resource model, meanwhile, posits that intimate partnerships involve diverse emotional, affective, economic, material, and care exchanges that are crucial to sustaining older adults’ mental health (Umberson et al., 2013). Finally, identity theory suggests that the symbolic status attached to couple relationships means that partnerships boost mental health by providing social recognition and identity affirmation (Dush and Amato, 2005; Evans et al., 2023). Our research is motivated by these frameworks, which collectively highlight the importance of intimate partnerships in supporting successful aging, and we extend and enrich existing theories by examining the implications of family diversity, particularly the understudied partnership form of LAT, for older adults’ mental health.

Because LAT, cohabitation, and marriage represent distinct types of social convoy, sustain different kinds and levels of resource exchange, and enjoy differential social recognition, their mental health benefits for older adults are likely to vary. Previous evidence from countries such as Australia, Germany, and the United States shows that the transition from singlehood to LAT is associated with mental health benefits, and compared with cohabiting and married couples, LAT couples enjoy a similar sense of companionship and a comparable level of emotional support but have a lower level of material and care exchange (Evans et al., 2023; Ševčíková et al., 2021; Strohm et al., 2009; Wu and Brown, 2022). The latter result is not entirely surprising because not living in the same household means that LAT couples may not be engaged in intense day-to-day interactions and exchanges at close quarters; conversely, those who prefer to avoid intense exchanges and care commitments may also prefer LAT. Moreover, the rise of unmarried cohabitation among older adults in the past couple of decades has been accompanied by increased recognition that cohabitation is a normative partnership type alongside marriage (Brown et al., 2022; Manning and Brown, 2011). In contrast, social recognition of LAT relationships still lags far behind, with LAT couples still often subjected to negative perceptions or stigma (Kobayashi et al., 2017). This may be especially relevant in later life; as compared with their younger counterparts, older adults may be more likely to endorse traditional and well-institutionalized partnership types such as marriage as opposed to LAT. Over and above LAT and cohabitation, marriage also confers on older adults legal and institutional protections, which may entail additional mental health benefits. In sum, existing theories and research suggest that compared with singlehood, LAT may be associated with mental health benefits for older adults, but to a lesser extent than cohabitation and particularly marriage.

### Partnership Types and Mental Strain

The mental health benefits of couple relationships are often accompanied by mental strain associated partly with negotiations and conflicts between partners (Broese van Groenou et al., 2019; Duncan, 2015; Evans et al., 2023). Compared with LAT partners residing in separate households, cohabiting partners living in the same household—whether married or unmarried—often experience intense negotiations of everyday routines and frictions at close quarters (Ševčíková et al., 2021; Yucel and Latshaw, 2023). Mental strain can also arise from co-residential partners’ perceived obligations and commitments to each other. Particularly for older adults, their often more intense care needs, coupled with expectations that a co-residential or married partner will help address these, mean that normative expectations to be (legally) liable for and to take care of each other may place a greater strain on cohabiting and particularly married older adults than on their LAT counterparts (Ghazanfareeon Karlsson and Espvall, 2016; Upton-Davis, 2012). Furthermore, legal obligations and institutional binding associated with marriage make it more difficult and costly for older adults to exit an unhappy marriage than an unhappy LAT relationship (Lewin, 2017; Upton-Davis, 2012). Because LAT often reflects older people’s preference to balance intimacy and companionship with personal space and autonomy (Connidis et al., 2017; Coulter and Hu, 2017; Karlsson and Borell, 2002), the personal space and autonomy afforded by LAT may buffer potential conflicts between partners and thus help to reduce mental strain. Existing theories and research, therefore, suggest that LAT may be associated with a lower level of mental strain compared with cohabitation and marriage (Lewin, 2017).

As individuals transition out of couple relationships, events such as moving out, dividing shared property, divorce proceedings, and the death of a partner are all key stressors that substantially heighten partners’ mental strain; such strain can be particularly stressful for older adults given the often-lengthy duration of their partnerships and their heightened vulnerability to the loss of a partner (Lin et al., 2019). As exiting a LAT relationship is usually less stressful and is also associated with lower economic and emotional costs (Lewin, 2017), the negative mental health ramifications of exiting a partnership may be smaller for older adults in LAT relationships than for those in cohabitation and marriage.

### Gender Differences

Bernard’s (1982) seminal work on “his” and “her” marriages suggests that women and men experience couple relationships in different ways. This insight has since inspired decades of research into gender differences in partners’ mental health, although as yet such work has seldom considered LAT relationships. Existing research suggests that men, particularly in marriage, tend to enjoy a higher level of relationship well-being and satisfaction than women (Dush and Amato, 2005; Lin et al., 2019; Umberson et al., 2013). Such gender differences arise partly from the traditional gender division of domestic and care labor (Umberson et al., 2013), which tends to be more closely endorsed by older rather than younger people in the United Kingdom (Perales et al., 2019). There are several reasons to expect LAT, as well as moving into and out of a LAT relationship, to also relate to older adults’ mental health in a gendered fashion.

In married and cohabiting unions, older women typically undertake a larger share of domestic and care tasks than older men (Noël-Miller, 2010; Spitze and Ward, 2000). Indeed, research shows that older men’s entry into a couple relationship is often (partly) driven by their pragmatic care demands, whereas older women are more prone to pursue emotional closeness and companionship (Broese van Groenou et al., 2019; Lin et al., 2019). In the United Kingdom, the gender gap in domestic labor in cohabiting and married couples is particularly pronounced among older adults (Kil et al., 2016). By contrast, LAT may afford older women the personal space, autonomy, and agency to avoid the often-onerous care commitments and domestic responsibilities associated with cohabitation and marriage (Duncan, 2015; Upton-Davis, 2015). Additionally, as compared with men, the highly gendered care responsibilities for other family members (e.g., grandchildren) placed on women in later life mean that LAT provides a more viable and less stressful means of repartnering for them to manage preexisting family relations and obligations (Connidis et al., 2017; Karlsson & Borell, 2002; Upton-Davis, 2015). Indeed, older women are more likely than men to care for grandchildren in the United Kingdom (Bordone et al., 2020), particularly given the country’s austerity policies and retrenchment of child welfare support since 2010 (Karamessini and Rubery, 2013). In contrast, older men in LAT relationships may be less able to receive (intense) care and domestic provision from their partners compared with those in cohabitation or marriage (Broese van Groenou et al., 2019). In sum, older women may have more to gain than older men from LAT (as opposed to marriage and cohabitation) due to the greater agency, autonomy, and freedom LAT relationships provide.

### Hypotheses

In light of the earlier discussion, this study tests three hypotheses. First, should LAT bring about mental health benefits compared with singlehood, Hypothesis 1A will hold; should the mental health gains outweigh the mental strain to a greater extent in marriage and cohabitation than in LAT, Hypothesis 1B will hold. However, we may not find empirical support for Hypothesis 1B because although LAT conveys fewer mental health benefits compared with marriage and cohabitation, it may also entail lesser mental strain. Second, focusing specifically on the transition out of couple relationships, we expect the mental health implications of exiting LAT versus marriage and cohabitation to vary according to Hypothesis 2. Finally, Hypothesis 3 specifies our expected gender differences in the mental health benefits of LAT.

**Hypothesis 1**: Compared with singlehood (never married, widowed, divorced/separated), being in and moving into a LAT relationship are associated with better mental health among older adults (H1A), but to a lesser extent than being in and moving into cohabiting and marital relationships (H1B).

**Hypothesis 2:** Exiting LAT relationships is associated with a smaller mental health decline than exiting cohabitation and marriage.

**Hypothesis 3**: The mental health benefits of LAT are greater for women than for men.

## Method

### Data and Sample

We used nationally representative data from the UKHLS. Initiated in 2009, the UKHLS interviewed around 50,000 individuals aged 16 and over from 30,000 households in its first wave, with respondents tracked and reinterviewed annually (Buck and McFall, 2012). The survey had a household response rate of 57% in its first wave, with this increasing to over 80% as the survey became established over subsequent waves, which compares well with many large-scale, population-wide panel surveys; rates of attrition in UKHLS are also relatively low, with longitudinal reinterview rates exceeding 80% after Wave 3—the first data wave included in our study (www.understandingsociety.ac.uk/documentation/user-guides). Moreover, attrition is generally lower among older than younger adults (Cabrera-Álvarez et al., 2023). The UKHLS adopts a complex mixed-mode design combining face-to-face computer-assisted personal interviews (which include a dedicated self-completion module) and computer-assisted telephone interviews and web questionnaire returns incorporating the same questions. Questions on mental health and LAT (from Wave 3 onwards) are asked in the self-completion module delivered to all online and telephone respondents and completed by the vast majority of face-to-face respondents (Buck and McFall, 2012). Because we also measure people’s transition in and out of LAT, we primarily use data from Wave 4 (2012–2014) to the latest, Wave 13 (2021–2023), with data from Waves 1–3 used mostly for imputing time-invariant measures.

To construct our analytical sample, we limited the sample to those who participated in the self-completion module (*N* = 326,064 person–waves, 55,874 persons). Following the definition of older adults adopted by the United Nations (2024), we limited our sample to person–wave observations aged 60 or over (*N* = 104,332 person–waves, 18,341 persons); we further deleted fewer than one percent of person–waves aged over 85 to minimize mortality bias (*N* = 99,424 person–waves, 17,738 persons). This means that our sample includes respondents who reach 60 years of age during our observation period, and it excludes respondents as they age beyond 85. We then listwise deleted 3.3% of person–waves with missing data (*N* = 96,157 person–waves, 17,509 persons). After excluding older adults observed only once (necessary for fixed effects estimations), our final analytical sample contains 93,885 observations of 15,237 respondents aged between 60 and 85. The respondents were observed for an average of 6.2 waves, and around 19% were observed across all waves. We present the sample characteristics in Table 1 and step-by-step information on analytical sample construction in Supplementary Tables S1 and S2.

**Table 1.** Sample Characteristics

Variable	Minimum	Maximum	Mean/Proportion (*SD*)		Gender difference (*p*)
			Women	Men	
SF-12 MCS (mental well-being, high = good)^a^	–5.44	2.85	–0.08	0.10	<.001
			(1.04)	(0.95)	
GHQ-12 (mental distress, high = poor)^a^	–2.14	5.19	0.11	–0.13	<.001
			(1.04)	(0.93)	
Age	60.00	85.00	69.80	69.90	.016
			(6.65)	(6.56)	
Monthly gross income (£1,000)	0	11.50	1.62	2.53	<.001
			(1.25)	(1.87)	
Self-reported health (high = good)	1.00	5.00	3.06	3.07	.142
			(1.06)	(1.05)	
Limited ADLs (1 = yes)	0	1	0.52	0.51	.178
Live alone (1 = yes)	0	1	0.32	0.20	<.001
Live with parent(s) (1 = yes)	0	1	0.01	0.01	.957
Live with son(s)/daughter(s) (1 = yes)	0	1	0.13	0.15	<.001
COVID restrictions (1 = yes)	0	1	0.22	0.22	.593
*N* (person–year)			50,805	43,080	
*N* (person)			8,253	6,984	

*Notes*: ADLs = Activities of daily living; COVID = coronavirus disease 2019; GHQ-12 = 12-Item General Health Questionnaire (Likert score); *SD* = Standard deviations for continuous variables are presented in parentheses; SF-12 MCS = 12-Item Short Form Survey Mental Component Summary. Dummy variables have a minimum value of 0 and a maximum value of 1. Two-tailed tests for gender differences. Observations are broadly evenly distributed across Waves 4–13 of the UKHLS (2012–2023).

^a^Standardized in the full (women + men) sample.

### Measures

#### Mental health

We captured respondents’ mental health using two sets of measures. First, we used the mental component score of the 12-Item Short Form Survey (SF-12 MCS) to measure positive mental well-being (Jakobsson, 2007). Second, we used the 12-item General Health Questionnaire 36-point Likert scale (GHQ-12) to measure respondents’ mental distress (Jackson, 2007). All our results are robust to using the GHQ-12 caseness scale. Further information on the wording of the mental health measures and construction of the mental health scales is presented in Supplementary Table S3.

#### Partnership types and transitions

For each person–wave observation, we distinguished between three partnership types: (a) LAT, (b) marriage, and (c) unmarried cohabitation, in addition to (d) singlehood. To measure LAT, the UKHLS asked respondents with no coresiding partner/spouse living in the same household to provide a “yes” or “no” answer to the question, “Do you have a steady relationship with someone you are not living with here, whom you think of as your ‘partner’? Please include your spouse or partner if you are not currently living with them.” For the 0.5% of observations for married respondents not living with their spouses, we classified them as LAT. Our robustness checks show that excluding these respondents from our analysis yields substantively consistent results (Supplementary Tables S4 and S5). Notably, the single group covers older adults who are never married, widowed, and divorced/separated, which is representative of the older population without a partner/spouse. This operationalization aligns with our focus on how having an intimate partner and the partner’s residential status shape older adults’ mental health. We created dummy variables to capture respondents’ transitions in and out of each partnership type between waves *t* – 1 and *t*. By cross-tabulating respondents’ partnership types at *t* – 1 and 1, we created further dummy variables capturing respondents’ detailed partnership-type transitions.

#### Control variables

As we used fixed effects models, we only included time-variant controls that may confound the relationship between older adults’ partnership type and mental health. We controlled for respondents’ age and its quadratic term (Evans et al., 2023). To account for respondents’ socioeconomic status (Connidis et al., 2017), we controlled for individual gross monthly income before tax, which comprehensively captures income from all sources, including wages, pensions, investments, etc. We did not control for education because older adults’ qualifications seldom change across waves. We controlled for older adults’ self-reported health measured using a 5-point Likert scale (high = good) and whether they were limited in their activities of daily living (0 = no, 1 = yes), which may shape both their intimate relationships and mental health (Umberson et al., 2013). We used a dummy variable to capture living alone, as this is often associated with loneliness and poor mental health among older adults (de Jong Gierveld and Broese van Groenou, 2016). We controlled for whether one lived with one’s parent(s) and children (both biological and non-biological), respectively (Lewin, 2017; Yucel and Latshaw, 2023). We also included survey wave dummies in all models. Finally, because the COVID-19 pandemic severely curtailed interhousehold mixing and adversely affected older adults’ mental health (Hu & Qian, 2021), we used a dummy variable to capture whether any COVID-19 restriction (e.g., lockdown, social distancing, self-isolation, etc.) was in place on the exact day when each respondent completed the survey (Mathieu et al., 2020). Early in our research, we also experimented with controlling for older adults’ employment status, the age of the youngest co-residing child under 16, urban (vs rural) residence, housing tenure (owner, social renter, private render), duration of the current relationship, the number of previous marriages, and a measure distinguishing different types of singlehood (never married, widowed, divorced/separated). Because the inclusion of these variables neither affected the relationship between partnership types/transitions and mental health nor improved model fit, they were excluded from the final models (full details available upon request).

### Analytical Strategy

We conducted our analysis in three steps. First, we used descriptive statistics to provide a national picture of family diversity among older adults in the United Kingdom, covering the prevalence of partnership types among older adults and their transitions into and out of each partnership type between *t* – 1 and *t*. Second, we estimated fixed effects models to examine how older adults’ mental health varied with their partnership types. Finally, we estimated fixed effects models to assess how older adults’ mental health varied with their transitions into and out of each partnership type. The fixed effects models account for both observed and unobserved time-invariant individual characteristics, which enables the examination of how within-person changes in partnership type relate to the changes in the same older adults’ mental health (Allison, 2009). Because the same individuals were observed multiple times in our sample, we clustered standard errors within individuals (Abadie et al., 2022). We conducted all analyses separately for women and men, and we conducted postestimation tests for gender differences in the fixed effects model coefficients by reestimating all models using the pooled (women + men) sample and including interaction terms between gender and our focal predictors (see Supplementary Tables S6 and S7 for the interaction results).

## Results

### Descriptive Results

Table 2 describes the distributions of older adults’ partnership types and changes between *t* – 1 and *t*. The results show that a similar 3%–4% of older women and men are in a LAT relationship in the United Kingdom, which is broadly comparable to the rates (4%–5%) of unmarried cohabitation in this population. Marriage is the most common partnership type among older U.K. adults—more so for men (72.7%) than women (57.6%) (*p*_difference_* < *0.001). In contrast, older women (35.4%) are nearly twice as likely as older men (18.2%) to be single (*p*_difference_ *<* 0.001).

**Table 2.** Distributions of Transitions in Partnership Type Between *T* – 1 and *T*

Partnership type at *t – *1	% of all observations moving into partnership type in the first column from *t *–** 1 to *t*	% of all observations moving out of partnership type in the first column from *t* – **1 to *t*	Partnership type at *t* (row %)			
			→ LAT	→ Married	→ Cohabit	→ Single
Women						
LAT (1,510; 3.0%)	0.9	0.9	71.5	2.9	2.3	23.4
Married (29,238; 57.6%)	0.4	1.4	0.2	97.6	0.2	2.0
Cohabit (2,094; 4.1%)	0.3	0.4	0.2	6.1	90.1	3.6
Single (17,963; 35.4%)	2.0	0.9	2.0	0.2	0.2	97.6
Men						
LAT (1,712; 4.0%)	1.3	1.1	71.2	3.0	2.7	23.1
Married (31,325; 72.7%)	0.6	1.0	0.3	98.6	0.2	0.9
Cohabit (2,204; 5.1%)	0.3	0.5	0.8	6.8	90.5	2.0
Single (7,839; 18.2%)	1.7	1.2	5.8	0.6	0.3	93.3

*Notes*: LAT = Living apart together. Numbers in parentheses indicate counts and column percentages at *t* – 1, which may not add up to 100% due to rounding (*n*; %). Cells in result columns 3–6 include row percentages based on the cross-tabulation of relationship status at *t* – 1 (listed in the first column of the table) and *t* (subheaders under “Partnership type at *t*”), which may not add up to 100% due to rounding. The single category covers those without a partner/spouse, including the never married, widowed, and divorced/separated.

The results in Table 2 also reveal considerable dynamism in older adults’ partnerships from one year to the next. For both women and men, around 29% of those in a LAT relationship at *t* – 1 exited the relationship at *t*, most often into singlehood (23% of cases) but also less frequently to marriage or cohabitation (2%–3%). Around 2% of women and 1% of men exited their marriages between *t* – 1 and *t*, with most moving into singlehood but some moving into LAT and cohabitation. During our observation period, about 10% of U.K. older adults, similarly among women and men, moved out of a cohabiting relationship, with most moving into a marriage. Notably, most older adults who transitioned out of singlehood moved into LAT as opposed to marriage and cohabitation. From *t – *1 to *t*, 2% and 6% of single older women and men (*p*_difference_ < 0.001), respectively, moved into a LAT union. The descriptive results suggest that LAT is an important partnership type among older adults in the United Kingdom, particularly for singles establishing a new partnership.

### Fixed Effects Regression Results

Table 3 presents the results from fixed effects models estimating how older adults’ mental health varies with partnership type. To facilitate the interpretation of model results, we standardized the dependent variables based on the full sample of both women and men. The results support Hypothesis 1A that LAT is associated with better mental health among older women and men compared with singlehood. Older women have a lower level of mental well-being (*B* = –0.088, *p* = .020) and a higher level of mental distress (*B* = 0.085, *p* = .034) when single than when in a LAT union. Similar results are observed for older men, whose mental health is worse—with a lower SF-12 MCS score (*B* = –0.165, *p* < .001) and a higher GHQ-12 score (*B* = 0.146, *p* < .001)—when single than when in a LAT relationship.

**Table 3.** Fixed Effects Models Predicting Older Adults’ Mental Health

Variable	Women		Men	
	SF-12 MCS (mental well-being)	GHQ-12 (mental distress)	SF-12 MCS (mental well-being)	GHQ-12 (mental distress)
Partnership type (ref. = LAT)				
Married	0.043	–0.037	0.031	–0.010
	(0.053)	(0.055)	(0.054)	(0.051)
Cohabit	0.096	–0.117^+^	0.062	–0.045
	(0.067)	(0.071)	(0.062)	(0.059)
Single	–0.088*	0.085*	–0.165***	0.146***
	(0.038)	(0.040)	(0.033)	(0.029)
Age	0.089***	–0.081**	0.144***	–0.113***
	(0.026)	(0.025)	(0.026)	(0.023)
Age (squared)	–0.000***	0.000***	–0.001***	0.001***
	(0.000)	(0.000)	(0.000)	(0.000)
Monthly gross income (£1,000)	–0.004	–0.001	0.006*	–0.013***
	(0.004)	(0.004)	(0.003)	(0.003)
Limited ADLs (ref. = no)	0.007	0.053***	–0.021*	0.040***
	(0.010)	(0.010)	(0.010)	(0.009)
Self-reported health	0.165***	–0.221***	0.134***	–0.203***
	(0.007)	(0.007)	(0.007)	(0.006)
Living alone (ref. = no)	–0.032	–0.015	–0.004	0.022
	(0.036)	(0.037)	(0.047)	(0.046)
Living with parent(s) (ref. = no)	–0.213*	0.073	–0.040	0.067
	(0.098)	(0.099)	(0.118)	(0.112)
Living with son(s)/daughter(s) (ref. = no)	–0.071**	0.047^+^	–0.046*	0.044*
	(0.026)	(0.026)	(0.022)	(0.021)
COVID restrictions (ref. = no)	–0.037^+^	0.111***	0.011	0.087***
	(0.021)	(0.021)	(0.021)	(0.020)
Person fixed effects	Yes	Yes	Yes	Yes
Wave fixed effects	Yes	Yes	Yes	Yes
Intercept	–4.439**	4.201**	–5.287***	4.077**
	(1.416)	(1.385)	(1.408)	(1.272)
*N* (person–wave)	50,805		43,080	
*N* (person)	8,253		6,984	
Within-*R*^2^ (without partnership type)	0.019	0.037	0.019	0.046
Within-*R*^2^ (with partnership type)	0.020	0.038	0.020	0.047

*Notes*: ADLs = Activities of daily living; COVID = coronavirus disease 2019; GHQ-12 = 12-Item General Health Questionnaire (Likert score); LAT = Living apart together; ref. = Reference category; SF-12 MCS = 12-Item Short Form Survey Mental Component Summary. Coefficients with robust standard errors in parentheses. None of the gender differences in the coefficients for partnership type is statistically significant at the 10% level or below—see Supplementary Table S6 for detailed test results. The single category covers those without a partner/spouse, including the never married, widowed, and divorced/separated.

^+^ *p* < .10.

*p < .05.

**p < .01.

***p < .001.

Nevertheless, Hypothesis 1B, that LAT is associated with lesser mental health benefits compared with marriage and cohabitation, has not received consistent support. The results show that older adults have similar levels of mental well-being (SF-12 MCS) and mental distress (GHQ-12) in LAT versus married and cohabiting relationships, for women and men alike. The only exception is that older women have a marginally lower level of mental distress when in a cohabiting as opposed to a LAT relationship (*B* = –0.117, *p* = .099). The results do not support Hypothesis 3 on gender differences, as none of the differences in the coefficients between women and men is statistically significant at the 10% level (see Supplementary Table S6 for detailed results).

Table 4 presents the average marginal effects (AME) from fixed effects models estimating how transitions into and out of each partnership type relate to older adults’ mental health, holding all control variables at their observed values. As shown in the first two result columns in Table 4, overall transitions into and out of LAT are not associated with older women’s mental health, and they are only associated with marginal mental health changes among older men. These gender differences are not statistically significant at the 10% level. As older men move out of LAT, they experience a 0.065-standard-deviation (*SD*) decrease in mental well-being (*p* = .085); as older men move into LAT, they experience a 0.053-*SD* decrease in mental distress (*p* = .078).

**Table 4.** Average Marginal Effects of Transitions in Partnership Type Between *T* – 1 and *T* on Older Adults’ Mental Health, From Fixed Effects Models

Partnership type at *t* – 1	All moving into partnership type in the first column from *t* – 1 to *t*	All moving out of partnership type in the first column from *t* – 1 to *t*	Partnership type at *t*			
			→ LAT	→ Married	→ Cohabit	→ Single
Women: SF-12 MCS (mental well-being)						
LAT	0.028	–0.008		0.030^a^	0.271^+^	–0.040
	(0.043)	(0.045)		(0.120)	(0.150)	(0.050)
Married	0.035	–0.546***^,^^a^	–0.303*		0.165^+^	–0.639***^,^^a^
	(0.055)	(0.039)	(0.125)		(0.097)	(0.043)
Cohabit	0.185**	–0.123*	–0.089	0.044		–0.399***
	(0.070)	(0.061)	(0.405)	(0.068)		(0.110)
Single	–0.418***^,^^a^	0.087*	0.092*	0.014	0.124	
	(0.033)	(0.041)	(0.045)	(0.138)	(0.134)	
Women: GHQ-12 (mental distress)						
LAT	–0.020	0.002		–0.175	–0.220	0.046
	(0.044)	(0.045)		(0.115)	(0.176)	(0.050)
Married	–0.028	0.609***^,^^a^	0.399***^,^^a^		–0.159^+^	0.703***^,^^a^
	(0.058)	(0.042)	(0.117)		(0.094)	(0.046)
Cohabit	–0.160*	0.170*	0.123	–0.013		0.473***
	(0.078)	(0.067)	(0.302)	(0.076)		(0.122)
Single	0.463***^,^^a^	–0.083^+^	–0.100*	0.069	–0.094	
	(0.034)	(0.043)	(0.047)	(0.131)	(0.163)	
Men: SF-12 MCS (mental well-being)						
LAT	0.049	–0.065^+^		0.288**^,^^a^	0.100	–0.135**
	(0.035)	(0.037)		(0.096)	(0.088)	(0.044)
Married	0.043	–0.340***^,^^a^	–0.073		0.101	–0.489***^,^^a^
	(0.049)	(0.046)	(0.084)		(0.105)	(0.057)
Cohabit	0.068	–0.106†	–0.019	–0.052		–0.334*
	(0.064)	(0.057)	(0.165)	(0.065)		(0.147)
Single	–0.297***^,^^a^	0.066†	0.073^+^	0.070	–0.073	
	(0.035)	(0.036)	(0.038)	(0.112)	(0.141)	
Men: GHQ-12 (mental distress)						
LAT	–0.053^+^	0.031		–0.181^+^	–0.028	0.069^+^
	(0.030)	(0.035)		(0.096)	(0.096)	(0.040)
Married	–0.088^+^	0.340***^,^^a^	0.044^a^		–0.106	0.498***^,^^a^
	(0.049)	(0.049)	(0.092)		(0.101)	(0.061)
Cohabit	–0.079	0.025	0.067	–0.067		0.338*
	(0.064)	(0.056)	(0.150)	(0.064)		(0.141)
Single	0.264***^,^^a^	–0.074*	–0.074*	–0.047	–0.113	
	(0.035)	(0.030)	(0.032)	(0.118)	(0.149)	

*Notes*: GHQ-12 = 12-Item General Health Questionnaire (Likert score); LAT = Living apart together; SF-12 MCS = 12-Item Short Form Survey Mental Component Summary. Average marginal effects with robust standard errors in parentheses. All models include all control variables reported in Table 3, as well as person and wave fixed effects. For the transition matrix, the partnership types listed in the first column of the table are the transition origins and those presented in the subheaders under “Partnership type at *t*” are the transition destinations. The single category covers those without a partner/spouse, including the never married, widowed, and divorced/separated.

^a^Gender difference statistically significant at the 10% level or below—see Supplementary Table S7 for detailed test results.

^+^ *p* < .10.

*p < .05.

**p < .01.

***p < .001.

Results for the detailed transitions (result columns 3–6 in Table 4), however, support Hypothesis 1A regarding the mental health benefits associated with LAT over singlehood. Compared with singlehood, LAT is associated with mental health benefits for both older women and men. Moving from singlehood to LAT is associated with a 0.092-*SD* (*p* = .042) and 0.073-*SD* (*p* = .060) increase in mental well-being and a 0.100-*SD* (*p* = .033) and 0.074-*SD* (*p* = .020) decrease in mental distress for older women and men, respectively. Moving from LAT to singlehood is associated with a 0.135-*SD* decrease (*p* = .002) in mental well-being and a 0.069-*SD* increase (*p* = .084) in mental distress for older men, but it is not associated with statistically significant changes in older women’s mental health. None of the gender differences in the coefficients for these transitions between singlehood and LAT is statistically significant at the 10% level. Thus, Hypothesis 3, predicting the greater mental health benefits of LAT for women than for men, is not supported.

Compared with LAT, cohabitation only brings about limited additional mental health benefits among older adults. The transition from LAT to cohabitation and that from cohabitation to LAT are hardly associated with changes in older adults’ mental health, with only one exception: moving from LAT to cohabitation is associated with a marginal increase in older women’s mental well-being (AME = 0.271, *p* = .071). Hypothesis 1B, comparing the mental health implications of LAT and cohabitation, has, therefore, only received very limited support. The results do, however, support Hypothesis 1B that marriage confers additional mental health benefits on older women and men over and above LAT. Older women moving from marriage to LAT experience a sizable 0.303-*SD* decrease (*p* = .015) in mental well-being and a 0.399-*SD* increase (*p* = .001) in mental distress. Older men moving from LAT to marriage experience a notable increase in mental well-being (AME = 0.288, *p* = .003) and a marginal decrease in mental distress (AME = –0.181, *p* = .058). Whereas moving from LAT into marriage is associated with a greater mental health improvement for men than for women (AME_SF-12 MCS_ = 0.288 vs 0.030, *p*_difference_ = 0.099), moving from marriage into LAT is associated with a greater mental health decline for women than for men (AME_GHQ-12_ = 0.399 vs 0.044, *p*_difference_ = 0.022).

The results of detailed partnership transitions also support Hypothesis 2 that exiting LAT is associated with smaller mental health declines compared with exiting marriage and cohabitation. Results in the last column of Table 4 show that the AMEs of moving from LAT to singlehood are much smaller than the AMEs of moving from cohabitation and marriage to singlehood, similarly for women and men (see Supplementary Table S8 for results of statistical tests comparing the AMEs).

## Discussion and Conclusion

Our research provides the first nationally representative evidence on the implications of LAT (vs cohabitation and marriage) for older adults’ mental health in the United Kingdom. Going beyond most preexisting research using cross-sectional data (Brown et al., 2022; Ciritel, 2022; Dush and Amato, 2005; Lewin, 2017; Liefbroer et al., 2015; Ross, 1995; Tai et al., 2014; Yucel and Latshaw, 2023), our fixed effects analysis of longitudinal data helps mitigate endogenous selection of older adults into different relationship types based on their observed and unobserved time-invariant characteristics. Our findings make several contributions to the scholarship on family diversity and well-being in the aging population, as discussed subsequently.

First, our descriptive findings underscore LAT as a key form of family diversity and complexity among older adults. It adds to a growing body of international research highlighting diverse ways in which older adults practice intimate partnerships (Connidis et al., 2017; Evans et al., 2023; Manning and Brown, 2011; Ševčíková et al., 2021; Wu and Brown, 2022). In the United Kingdom, we find that LAT is approximately as prevalent as unmarried cohabitation in the aging population. The rate of LAT among older adults in the United Kingdom (3%–4%) is also comparable to that in other countries such as the United States, Netherlands, and Canada (Connidis et al., 2017). For older singles entering a couple relationship in the United Kingdom, LAT is by far the most common partnership destination. For single older women, entering LAT is 10 times more likely than marriage or cohabitation, and it is nearly 10 times more likely than marriage and about 20 times more likely than cohabitation for older men. These findings echo previous qualitative insights on older adults’ preference for LAT (Benson and Coleman, 2016) and emphasize the importance of understanding the implications of LAT for older adults’ mental health.

Second, fixed effects estimates reveal clear mental health benefits of LAT vis-à-vis singlehood among older adults in the United Kingdom, which chimes with an emerging body of evidence from the United States (Brown et al., 2022), Australia (Evans et al., 2023), and Europe (Rapp and Stauder, 2020; Ševčíková et al., 2021; Yucel and Latshaw, 2023). As LAT relationships stretch across households, LAT often sustains complex and frequent material, informational, emotional, affective, and care exchanges beyond the confines of a single dwelling that are crucial to successful aging (Rowe and Khan, 1998, 2015). Our findings bring to the fore the mental health benefits of these exchanges that are largely invisible in most household-centered research on family relations and population aging. They also highlight the need for research examining the nature and patterns of exchanges in older adults’ LAT partnerships and their interhousehold social convoy more broadly. In addition, our findings reveal that there is very little variation in older adults’ mental health across the partnership types of LAT, cohabitation, and marriage. Furthermore, existing qualitative evidence highlights the role played by LAT in providing older women with the opportunity to develop individual autonomy and exercise agency more so than in cohabitation and marriage (Duncan, 2015; Upton-Davis, 2015). Our findings reaffirm prior evidence that men seem to benefit more than women from marriage in their mental health (Dush and Amato, 2005; Lin et al., 2019; Umberson et al., 2013). Extending existing scholarship, however, our findings show little evidence that the mental health benefits of LAT differ between older women and men. Therefore, LAT seems to provide a gender-egalitarian way for older women and men to access the mental health benefits associated with couple relationships.

Third, our analysis of detailed transitions into and out of distinct partnership types reveals important nuance in how the mental health implications of LAT differ from those of cohabitation and marriage. As older adults’ transition from LAT to cohabitation is hardly associated with any mental health benefits, our findings reflect critically on the long-held ideal of coresidence in couple relationships and its role in sustaining older adults’ mental health. The absence of evidence here could be because moving in with one’s partner entails both mental health benefits and heightened mental strain as partners come to terms with and negotiate changes in everyday routines at close quarters. In addition, echoing previous evidence on the well-being premium associated with marriage (Broese van Groenou et al., 2019; Yucel and Latshaw, 2023), we find that moving from LAT to marriage is associated with improvements in older men’s—but not older women’s—mental health. Finally, our findings highlight that compared with moving out of LAT relationships, exiting cohabitation and particularly marriage is associated with much greater mental health declines among older women and men. Compared with moving into marriage, moving into LAT is associated with fewer mental health benefits, but moving out of a LAT relationship is also associated with lesser mental health harms compared with the transition out of marriage. In essence, LAT appears to offer a balanced option for older adults seeking to enter a partnership: lesser gain in mental health but also less exposure to risk. Indeed, avoiding complex decoupling (e.g., moving out, dividing property, undergoing divorce proceedings) may be a major reason why many older adults prefer LAT as a long-term arrangement, particularly given the complex family relationships and responsibilities they often juggle in later life (Connidis et al., 2017; Coulter and Hu, 2017; Karlsson and Borell, 2002).

The limitations of our study suggest several important directions for future research. First, although our fixed effects models account for unobserved time-invariant characteristics, and despite our inclusion of time-varying controls, our analysis could be affected by additional time-varying factors. For example, the UKHLS did not collect detailed information on respondents’ LAT partners, nor has it traced whether one has the same LAT partner across survey waves. This means that in what we expect to be a small number of cases, we have not been able to control for (unobserved) changes in partner characteristics. This limitation urges future surveys to develop robust methods to collect data on crucial non-household members (e.g., LAT partners). Second, due to cell size considerations, it is also beyond our scope to conduct further heterogeneity analyses, including distinguishing the nature of LAT (e.g., transitory vs long-term) (Connidis et al., 2017; Coulter and Hu, 2017), comparing different types of singlehood (i.e., never married, widowed, divorced/separated) and disentangling different pathways leading into and out of LAT, cohabitation, and marriage (e.g., separation from vs the death of one’s partner). With a larger sample, future research could examine the implications of more detailed partnership changes for older adults’ mental health. Finally, future research could more specifically examine the underlying mechanisms—including relationship satisfaction, functional exchanges, and relationship strain—in how partnership types, particularly LAT, shape older adults’ mental health.

Notwithstanding its limitations, our research illustrates the value of going beyond the household when examining the intimate connections in older adults’ lives that matter so much for their health outcomes. Given their distinctive position in the life course, older adults may be dissuaded or prevented by complex life circumstances—including relationships with ex-spouses, (grand)children, and care needs and responsibilities—from moving in with or marrying their intimate partner. LAT among older adults can also arise in cases where one partner moves into an institutionalized residence (e.g., medical facility, care home) due to their care and medical needs, and as a result of increasingly complex international migration patterns that give rise to transnational families. For policymakers and practitioners, therefore, our findings underscore the importance of building strong interhousehold (intimate) bonds in sustaining older adults’ mental health, similarly for women and men. As LAT illustrates an under-recognized aspect of family diversity in the aging population, our study more broadly brings to the fore the imperative of mainstreaming family diversity as a crucial lens in both research on and service provision to the aging population.

## Supplementary Material

gbae192_suppl_Supplementary_Tables

## Data Availability

The data used in this research were made available through the U.K. Data Archive. The United Kingdom Household Longitudinal Study is an initiative funded by the ESRC and various Government Departments, with scientific leadership by the Institute for Social and Economic Research, University of Essex, and survey delivery by NatCen Social Research and Kantar Public. Neither the original collectors of the data nor the Archive bear any responsibility for the analyses or interpretations presented here. The study’s design and its analysis were not pre-registered.
