# Supplementary material for: Living Apart Together and Older Adults’ Mental Health in the United Kingdom
Source: J Gerontol B Psychol Sci Soc Sci. 2024 Dec 3;80(3):gbae192. doi: 10.1093/geronb/gbae192 (PMC11878551; doi:10.1093/geronb/gbae192)
Supplement: gbae192_suppl_Supplementary_Tables [file gbae192_suppl_supplementary_tables.docx]

***The Journals of Gerontology, Series B: Psychological Sciences and Social Sciences:*** **Supplementary Material: Hu & Coulter. Living Apart Together and Older Adults’ Mental Health in the United Kingdom.**

**Table S1.** Step-by-step information on analytical sample construction

**Table S2.** Comparing sample characteristics for older adults observed one or more times vs. observed at least twice

**Table S3.** Further information on mental health measures

**Table S4.** Results for the main effects of partnership type, excluding married LAT respondents

**Table S5.** Results for partnership transitions, excluding married LAT respondents

**Table S6.** Coefficients (with standard errors in parentheses) for gender interactions, results for gender differences in the main effects of partnership type (Table 3 in the main article)

**Table S7.** Coefficients (with standard errors in parentheses) for gender interactions, results for gender differences in partnership transitions (Table 4 in the main article)

**Table S8.** Statistical tests for differences in the AMEs of transitions into singlehood from LAT vs. from marriage and cohabitation

| **Table S1.** Step-by-step information on analytical sample construction | | |
| --- | --- | --- |
| **Step** | **Sample description/deletion** | **Sample remaining** |
| 0 | UKHLS full sample waves 4–13. | *N* = 378,221 person–waves, 66,510 persons |
| 1 | Limit the sample to those who participated in the self-completion module (including mental health and LAT status questions). | *N* = 326,064 person–waves, 55,874 persons |
| 2 | Delete those aged < 60 at the time of survey (using the United Nations definition of older adults). | *N* = 104,332 person–waves, 18,341 persons |
| 3 | Delete those aged > 85 at the time of survey (to minimize mortality bias). | *N* = 99,424 person–waves, 17,738 persons |
| 4 | Listwise deletion of missing values—person-wave deleted for each variable reported in the order listed below, with only 3.3% person–waves of the sample from the preceding step deleted:   - 891 for GHQ-12 - 1,058 for SF-12 MCS - 115 for relationship status in the current wave of data - 391 for relationship status in the previous wave of data (used for constructing relationship transitions) - 43 for limited activities of daily living - 77 for living with parent(s) - 692 for living alone | *N* = 96,157 person–waves, 17,509 persons |
| 5 | Delete older adults only observed once for fixed effects analysis (see Table S2 for further information on this deletion) | *N* = 93,885 person–waves, 15,237 persons (final analytical sample) |
| *Note*: UKHLS = United Kingdom Household Longitudinal Survey. LAT= Living apart together. GHQ-12 = 12-Item General Health Questionnaire (Likert score). MCS = 12-Item Short Form Survey Mental Component Summary. | | |

| **Table S2.** Comparing sample characteristics for older adults observed one or more times vs. observed at least twice | | | |
| --- | --- | --- | --- |
|  | Mean/proportion (SD) | | Between-sample differences *p* value for *t*/χ^2^ test |
|  | Older adults at least two observations | Older adults with one or more observations |  |
| SF-12 MCS (high = good, unstandardized) | 51.43 | 51.83 | 0.247 |
|  | (9.34) | (9.39) |  |
| GHQ-12 (high = poor, unstandardized) | 10.50 | 10.53 | 0.299 |
|  | (4.92) | (4.94) |  |
| Age | 69.84 | 69.80 | 0.184 |
|  | (6.61) | (6.68) |  |
| Monthly gross income (£1,000) | 2.04 | 2.04 | 0.879 |
|  | (1.63) | (1.63) |  |
| Self-reported health (high = good) | 3.07 | 3.06 | 0.155 |
|  | (1.06) | (1.06) |  |
| Limited ADLs (1 = yes) | 0.52 | 0.52 | 0.736 |
| Live alone (1 = yes) | 0.27 | 0.27 | 0.671 |
| Live with parent(s) (1 = yes) | 0.01 | 0.01 | 0.712 |
| Live with son(s)/daughter(s) (1 = yes) | 0.14 | 0.14 | 0.058 |
| COVID restrictions (1 = yes) | 0.22 | 0.22 | 0.410 |
| *N* (person–wave) | 93,885  (final analytical sample) | 96,157  (step-4 sample in Table S1) |  |
| *Note*: SD = Standard deviations for continuous variables. SF-12 MCS = 12-Item Short Form Survey Mental Component Summary. GHQ-12 = 12-Item General Health Questionnaire (Likert score). ADLs = Activities of daily living. Observations are broadly evenly distributed across waves 4 to 13 of the UKHLS (2012–2023), and no statistically significant differences (at the 5% level) is observed between the two samples. | | | |

| **Table S3.** Further information on mental health measures |
| --- |
| *Wording of mental health questions* |
| - SF-12 (12-Item Short Form Survey – Mental Component Summary): <https://www.understandingsociety.ac.uk/wp-content/uploads/documentation/main-survey/questionnaires/6614-main-survey-questionnaire-w14.pdf> (pages 773–777) - GHQ-12 (12-Item General Health Questionnaire): <https://www.understandingsociety.ac.uk/wp-content/uploads/documentation/main-survey/questionnaires/6614-main-survey-questionnaire-w14.pdf> (pages 777–783) |
| *Further information on the calculation of the mental health measures:* |
| - SF-12 (12-Item Short Form Survey – Mental Component Summary): <https://www.understandingsociety.ac.uk/documentation/mainstage/variables/sf12mcs_dv/> - GHQ-12 (12-Item General Health Questionnaire): <https://www.understandingsociety.ac.uk/documentation/mainstage/variables/scghq1_dv/> |

| **Table S4.** Results for the main effects of partnership type, excluding married LAT respondents | | | | |
| --- | --- | --- | --- | --- |
|  | Women | | Men | |
|  | SF-12 MCS | GHQ-12 | SF-12 MCS | GHQ-12 |
| Partnership type (ref. = LAT) |  |  |  |  |
| Married | –0.016 | 0.021 | 0.026 | 0.006 |
|  | (0.057) | (0.060) | (0.059) | (0.054) |
| Cohabit | 0.047 | –0.071 | 0.057 | –0.032 |
|  | (0.069) | (0.074) | (0.065) | (0.061) |
| Single | –0.129** | 0.128** | –0.163*** | 0.152*** |
|  | (0.040) | (0.043) | (0.034) | (0.030) |
| Control variables, including person and wave fixed effects | Yes | Yes | Yes | Yes |
| *Note*: Coefficients with robust standard errors in parentheses. ref. = reference category. LAT = Living apart together. SF-12 MCS = 12-Item Short Form Survey Mental Component Summary. GHQ-12 = 12-Item General Health Questionnaire (Likert score). ADLs = Activities of daily living. The single category covers those without a partner/spouse, including the never married, widowed, and divorced/separated. | | | | |
| ** *p* < 0.01, *** *p* < 0.001. | | | | |

| **Table S5.** Results for partnership transitions, excluding married LAT respondents | | | | | | | |
| --- | --- | --- | --- | --- | --- | --- | --- |
| Partnership  type at *t* – 1 | All moving into partnership type in the first column from  *t –* 1 to *t* | All moving  out of partnership type in the first column from  *t –* 1 to *t* | Partnership type at *t* | | | |  |
|  |  |  | → LAT | → Married | → Cohabit | → Single |  |
| Women: SF-12 MCS |  |  |  |  |  |  |  |
| LAT | 0.069 | –0.021 |  | –0.063 | 0.269+ | –0.045 |  |
|  | (0.045) | (0.045) |  | (0.122) | (0.150) | (0.050) |  |
| Married | 0.017 | –0.563*** | –0.395 |  | 0.165+ | –0.642*** |  |
|  | (0.055) | (0.041) | (0.282) |  | (0.097) | (0.043) |  |
| Cohabit | 0.185** | –0.123* | –0.090 | 0.043 |  | –0.400*** |  |
|  | (0.070) | (0.061) | (0.405) | (0.068) |  | (0.110) |  |
| Single | –0.423*** | 0.082* | 0.088+ | 0.005 | 0.126 |  |  |
|  | (0.033) | (0.041) | (0.046) | (0.138) | (0.134) |  |  |
| Women: GHQ-12 |  |  |  |  |  |  |  |
| LAT | –0.080+ | 0.010 |  | –0.097 | –0.220 | 0.044 |  |
|  | (0.047) | (0.046) |  | (0.128) | (0.177) | (0.050) |  |
| Married | –0.008 | 0.620*** | 0.223 |  | –0.160+ | 0.710*** |  |
|  | (0.059) | (0.044) | (0.301) |  | (0.094) | (0.046) |  |
| Cohabit | –0.162* | 0.171* | 0.124 | –0.013 |  | 0.477*** |  |
|  | (0.078) | (0.067) | (0.303) | (0.076) |  | (0.122) |  |
| Single | 0.468*** | –0.075+ | –0.092+ | 0.079 | –0.098 |  |  |
|  | (0.035) | (0.043) | (0.047) | (0.132) | (0.163) |  |  |
| Men: SF-12 MCS |  |  |  |  |  |  |  |
| LAT | 0.070+ | –0.056 |  | 0.325** | 0.098 | –0.125** |  |
|  | (0.037) | (0.038) |  | (0.108) | (0.088) | (0.044) |  |
| Married | 0.043 | –0.366*** | 0.144 |  | 0.101 | –0.488*** |  |
|  | (0.050) | (0.050) | (0.147) |  | (0.105) | (0.057) |  |
| Cohabit | 0.068 | –0.106+ | –0.014 | –0.052 |  | –0.330* |  |
|  | (0.064) | (0.058) | (0.168) | (0.065) |  | (0.148) |  |
| Single | –0.292*** | 0.062+ | 0.070+ | 0.058 | –0.074 |  |  |
|  | (0.035) | (0.036) | (0.039) | (0.114) | (0.141) |  |  |
| Men: GHQ-12 |  |  |  |  |  |  |  |
| LAT | –0.075* | 0.023 |  | –0.221* | –0.028 | 0.061 |  |
|  | (0.031) | (0.035) |  | (0.106) | (0.096) | (0.039) |  |
| Married | –0.095+ | 0.380*** | –0.018 |  | –0.106 | 0.498*** |  |
|  | (0.050) | (0.053) | (0.191) |  | (0.101) | (0.061) |  |
| Cohabit | –0.079 | 0.022 | 0.031 | –0.067 |  | 0.338* |  |
|  | (0.064) | (0.056) | (0.154) | (0.064) |  | (0.141) |  |
| Single | 0.262*** | –0.080** | –0.081* | –0.048 | –0.114 |  |  |
|  | (0.035) | (0.031) | (0.032) | (0.120) | (0.149) |  |  |

*Note*: Average marginal effects with robust standard errors in parentheses. LAT = Living apart together. SF-12 MCS = 12-Item Short Form Survey Mental Component Summary. GHQ-12 = 12-Item General Health Questionnaire (Likert score). All models include all control variables reported in Table 3, as well as person and wave fixed effects. For the transition matrix reported in shaded cells, the partnership types listed in the first column are the transition origins and those presented in the headers of the shaded columns are the transition destinations. The single category covers those without a partner/spouse, including the never married, widowed, and divorced/separated.

+ *p* < 0.10, * *p* < 0.05, ** *p* < 0.01, *** *p* < 0.001

| **Table S6.** Coefficients (with standard errors in parentheses) for gender interactions, results for gender differences in the main effects of partnership type (Table 3 in the main article) | | |
| --- | --- | --- |
|  | SF-12 MCS | GHQ-12 |
| Coefficients for partnership type × men (ref. = women), with with robust standard errors in parentheses | | |
| Partnership type (ref. = LAT) × men (ref. = women) |  |  |
| Married × men | –0.020 | –0.006 |
|  | (0.062) | (0.061) |
| Cohabit × men | –0.053 | 0.045 |
|  | (0.082) | (0.082) |
| Single × men | –0.079 | 0.062 |
|  | (0.050) | (0.049) |
| Main effects of gender | Yes | Yes |
| Main effects of partnership type | Yes | Yes |
| Control variables, including person and wave fixed effects | Yes | Yes |
| *Note*: *N* = 93,885 person–waves, 15,237 persons. ref. = reference category. LAT = Living apart together. SF-12 MCS = 12-Item Short Form Survey Mental Component Summary. GHQ-12 = 12-Item General Health Questionnaire (Likert score). None of the interaction effects is statistically significant at the 10% level. The single category covers those without a partner/spouse, including the never married, widowed, and divorced/separated. | | |

| **Table S7.** Coefficients (with standard errors in parentheses) for gender interactions, results for gender differences in partnership transitions (Table 4 in the main article) | | | | | | |
| --- | --- | --- | --- | --- | --- | --- |
| Partnership  type at *t* – 1 | All moving into partnership type in the first column from  *t –* 1 to *t* | All moving  out of partnership type in the first column from  *t –* 1 to *t* | Partnership type at *t* | | | |
|  |  |  | → LAT | → Married | → Cohabit | → Single |
| Coefficients for partnership transition × men (ref. = women), with with robust standard errors in parentheses | | | | | | |
| SF-12 MCS |  |  |  |  |  |  |
| LAT | 0.021 | –0.057 |  | 0.254+ | –0.174 | –0.095 |
|  | (0.055) | (0.058) |  | (0.154) | (0.173) | (0.066) |
| Married | 0.006 | 0.195*** | 0.224 |  | –0.073 | 0.142* |
|  | (0.074) | (0.059) | (0.149) |  | (0.143) | (0.069) |
| Cohabit | –0.122 | 0.017 | 0.058 | –0.096 |  | 0.070 |
|  | (0.094) | (0.084) | (0.436) | (0.094) |  | (0.184) |
| Single | 0.118* | –0.020 | –0.017 | 0.053 | –0.199 |  |
|  | (0.047) | (0.054) | (0.059) | (0.177) | (0.194) |  |
| GHQ-12 |  |  |  |  |  |  |
| LAT | –0.029 | 0.029 |  | –0.005 | 0.175 | 0.024 |
|  | (0.054) | (0.057) |  | (0.148) | (0.200) | (0.064) |
| Married | –0.059 | –0.239*** | –0.338* |  | 0.065 | –0.176* |
|  | (0.075) | (0.063) | (0.148) |  | (0.138) | (0.075) |
| Cohabit | 0.079 | –0.140 | –0.044 | –0.051 |  | –0.129 |
|  | (0.101) | (0.087) | (0.336) | (0.099) |  | (0.186) |
| Single | –0.182*** | 0.009 | 0.027 | –0.117 | –0.028 |  |
|  | (0.048) | (0.052) | (0.057) | (0.176) | (0.222) |  |
| *Note*: LAT = Living apart together. SF-12 MCS = 12-item Short Form Survey Mental Component Summary. GHQ-12 = 12-item General Health Questionnaire (Likert score). All models include all control variables reported in Table 3, the main effects of gender and partnership transition, as well as person and wave fixed effects. For the transition matrix reported in shaded cells, the partnership types listed in the first column are the transition origins and those presented in the headers of the shaded columns are the transition destinations. The single category covers those without a partner/spouse, including the never married, widowed, and divorced/separated. | | | | | | |
| * *p* < 0.05, ** *p* < 0.01, *** *p* < 0.001. | | | | | | |

| **Table S8.** Statistical tests for differences in the AMEs of transitions into singlehood from LAT vs. from marriage and cohabitation | | |
| --- | --- | --- |
|  | AME (SE) | Difference compared with reference AME (*p*) |
| Women: SF-12 MCS |  |  |
| LAT 🡪 single | –0.040 | Reference |
|  | (0.050) |  |
| Married 🡪 single | –0.639*** | < 0.001 |
|  | (0.043) |  |
| Cohabit 🡪 single | –0.399*** | 0.001 |
|  | (0.110) |  |
| Women: GHQ-12 |  |  |
| LAT 🡪 single | 0.046 | Reference |
|  | (0.050) |  |
| Married 🡪 single | 0.703*** | < 0.001 |
|  | (0.046) |  |
| Cohabit 🡪 single | 0.473*** | < 0.001 |
|  | (0.122) |  |
| Men: SF-12 MCS |  |  |
| LAT 🡪 single | –0.135** | Reference |
|  | (0.044) |  |
| Married 🡪 single | –0.489*** | < 0.001 |
|  | (0.057) |  |
| Cohabit 🡪 single | –0.334* | 0.157 |
|  | (0.147) |  |
| Men: GHQ-12 |  |  |
| LAT 🡪 single | 0.069+ | Reference |
|  | (0.040) |  |
| Married 🡪 single | 0.498*** | < 0.001 |
|  | (0.061) |  |
| Cohabit 🡪 single | 0.338* | 0.052 |
|  | (0.141) |  |
| *Note*: AME = Average marginal effects. SE = Robust standard errors. LAT = Living apart together. SF-12 MCS = 12-Item Short Form Survey Mental Component Summary. GHQ-12 = 12-Item General Health Questionnaire (Likert score). All models include all control variables reported in Table 3, as well as person and wave fixed effects. The single category covers those without a partner/spouse, including the never married, widowed, and divorced/separated. | | |
| + *p* < 0.10, * *p* < 0.05, ** *p* < 0.01, *** *p* < 0.001. |  |  |
